# Design of Anti-Angiogenic Peptidomimetics and Evaluation their Biological Activity by *In Vitro* Assays

**Published:** 2020

**Authors:** Mona Ghadam, Soroush Sardari, Mohammad Ali Shokrgozar, Mahdiyeh Sadat Mahdavi

**Affiliations:** 1. Department of Medical Biotechnology, Biotechnology Research Center, Pasteur Institute of Iran, Tehran, Iran; 2. National Cell Bank of Iran, Pasteur Institute of Iran, Tehran, Iran

**Keywords:** Angiogenesis Inhibitors, Drug design, Peptidomimetic, Vascular endothelial growth factor receptor

## Abstract

**Background:**

One of the important therapeutic approaches in cancer field is development of compounds which can block the initial tumor growth and the progression of tumor metastasis with no side effects. Thus, the recent study was carried out to design anti-VEGFR2-peptidomimetics as the most significant factor of angiogenesis process- and evaluate their biological activity by *in vitro* assays.

**Methods:**

We designed anti-VEGFR2 peptidomimetics with anti-angiogenic activity, including compound P (lactam derivative) and compound T (indole derivative) by using in silico methods. Then, the inhibitory activity on angiogenesis was evaluated by using angiogenesis specific assays such as Human Umbilical Vein Endothelial Cell (HUVEC) proliferation, tube formation in Matrigel, MTT and Real-Time PCR. IC50 values of the compounds were also determined by cytotoxicity plot in MTT assay.

**Results:**

Compounds P and T inhibited HUVEC cell proliferation and viability in a dose-dependent manner. The IC50 for compound T and compound P in HUVEC cell line were 113 and 115 *μg/ml*, respectively. Tube formation assay revealed that both compounds can inhibit angiogenesis effectively. The results of Real-Time PCR also showed these compounds are able to inhibit the expression of *CD31* gene in HUVEC cell line.

**Conclusion:**

Our study suggested that compounds P and T may act as therapeutic molecules, or lead compounds for development of angiogenesis inhibitors in VEGF-related diseases.

## Introduction

Angiogenesis or development of blood vessels 1,2 happens in physiological conditions such as wound healing. This process is resulted through coordination of different pro- and anti-angiogenic factors 3. In other words, the balance among these growth factors is missed in pathological conditions like rheumatoid arthritis, and especially cancers 4,5. Among these factors, Vascular Endothelial Growth Factor (VEGF) has gained a reputation for its outstanding role in angiogenesis. In fact, the interaction between VEGF and KDR (VEGF receptor-2) is considered as the most significant part in the process 6–11.

Several chemical or molecular approaches have been suggested for targeting VEGF-VEGF receptor interaction- such as ATP mimetics, tyrosine kinase inhibitors, antibodies, peptides and receptor blocking agents 12–15. In the area of peptides, ST100 and LPPHSS have been identified as anti-VEGFR2 peptides 7,16. Furthermore, some peptides have been identified, which inhibit the binding of VEGF to its receptors in Human Umbilical Vein Endothelial Cells (HUVECs) 17. Peptides are cost-effective and safe compared with monoclonal antibodies 18–20. Peptides have several advantages- such as high penetration into tissues and increased bioavailability 19. However, these peptides are faced with some limitations- such as high susceptibility to proteasomal degradation 20. As an alternative, pseudo peptides and modified peptides that are used as peptidomimetics. The resistance of peptidomimetics to proteasomal degradation is higher than peptides, which results in increase of peptidomimetics bioavailability 21–24. Thus, peptidomimetics are considered as suitable candidates for new generation of therapeutic compounds, which have a variety of advantages- such as being water soluble, non-immunogenic, increased selectivity, capability to cross tissue barriers, decreased side effects and toxicity compared with peptides 25.

Design of peptidomimetics and evaluation their biological activity have been reported by various research groups. For instance, Moradi *et al*, designed antifungal indole and pyrrolidine-2, 4-dione derivative peptidomimetic against *Aspergillus niger*, *Candida albicans*, and *Saccharomyces cerevisiae* fungi. The authors observed the structure C2 has a potent antifungal activity and could be used as a template for designing anti-fungal peptidomemetics.

Computation and bioinformatics has become a key aspect of drug discovery and contributing to both target discovery and validation. Bioinformatics will continue to play an important role in response to the waves of genome-wide data sources- including Expressed Sequence Tags (ESTs), microbial genome sequences, model organism sequences, polymorphisms, gene expression data and proteomics 26. However, such knowledge sources must be integrated in future. The bioinformatics tools can be used to discover the peptidomimetics 26,27. Among these tools, Super-Mimic software identifies compounds that mimic parts of a protein. In a short statement, Super-Mimic provides libraries that contain peptidomimetic building blocks in one hand and protein structures on the other hand. The search for promising peptidomimetics for target peptide is based on the superposition of the peptide with several conformers of the mimetic. This search results in a list of peptidemimetics, the position within the protein where the mimetic could be inserted, and also the conformation of the mimetic that fits the best 26.

Since, tumor growth, progression, and metastasis are severely influenced by generation of pro-angiogenic VEGF, promising anti-angiogenic drugs are important and currently available; however, their susceptibilities to drug resistance and long term toxicity are serious obstacles to their use. As a result, we require the development of novel therapeutic approaches for effective and safe angiogenic inhibitors. The current study, was carried out to design anti-VEGFR peptidomimetics and evaluate their biological activity by *in vitro* assays- such as tube formation, HUVEC proliferation and the gene expression of CD31 (Real-Time PCR) in HUVEC cell line.

## Materials and Methods

### Collection of anti-angiogenesis peptides

In the current study, several anti-VEGFR2 peptides were collected from previous studies (Table 1), and their amino acid sequences were used as the input data for sequence alignment and to identify the common amino acid sequences among them 7,28–32.

**Table 1.**
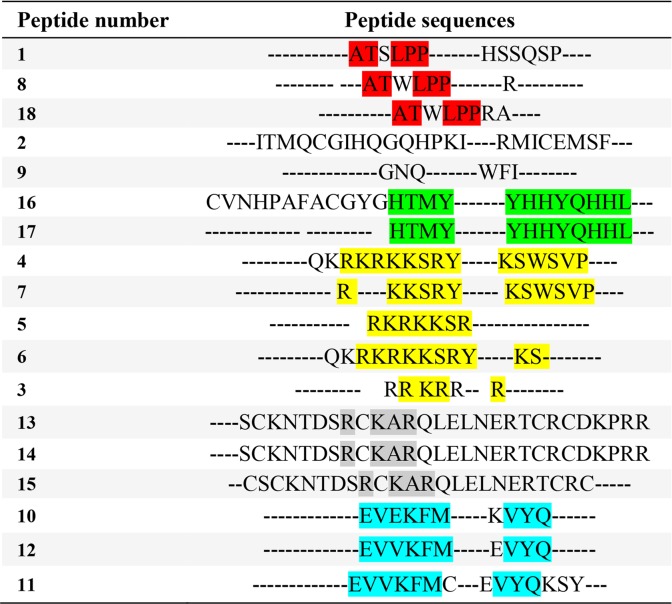
The result of multiple sequence alignment of anti-VEGFR2 peptides (Binétruy-Tournaire *et al*, Vicari *et al*, Selwood *et al*, Kim *et al*, Garcia-Aranda *et al*)

### Sequence alignments

T-Coffee V 5.13, multiple sequence alignment tool, was employed for sequence alignment of anti-VEGFR2 peptides 33. In this way, the various sets of sequence alignments were carried out in order to find a better homology among anti-VEGFR2 peptide sequences. After sequence alignment, peptides with more homology in sequence were selected to extract the final patterns among each group (Table 1). In the following, peptidomimetic structures were designed based on the final pattern obtained from sequence alignment using T-coffee, in which, scores are based on colors; positions that have no consistency with the in-house peptide library are in blue, a little in green, better positions in red, and finally yellow. The Basic Local Alignment Search Tool (BLAST) was used to discover final pattern from sequences alignments.

### Mimetic design

**Figure 1.**
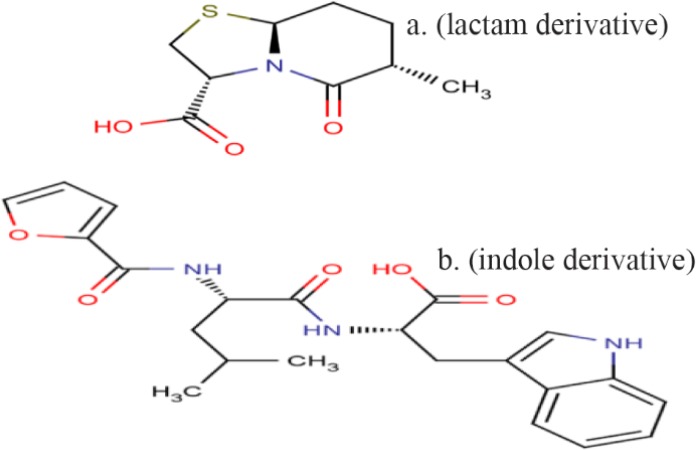
The structure of compound P (lactam derivative) and compound T (indole derivative).

SuperMimic software provides a library of peptidomimetic structures which have been arranged in sub-libraries such as beta-turn or gamma-turn mimetics. In this study, SuperMimic was used to replace RKRKKSR with peptidomimetics structures which are in SuperMimic library. Among designed peptidomimetics, two structures were selected with lower than 9×10^−3^ Å Root Mean Square Deviation (RMSD) of the backbone atoms and more similarity in chemical nature to the backbone and their side chains (Figure 1) 26.

### Drug preparation and dilution

Compound P and compound T were purchased from Jaber-ibn-Hayan and Sigma companies, respectively. These two compounds were diluted in water and DMEM medium at concentrations range of 25, 50, 100, 200, and 400 *μg/ml*.

### Cells culture condition and reagents

Adherent cell line of HUVEC was provided from Department of Cell Bank, Pasteur Institute of Iran. The cells were cultured in media consisting Dulbecco’s Modified Eagles Medium (DMEM; Invitrogen, Carlsbad, CA) with 10% Fetal Bovine Serum (FBS), penicillin (100 *U/ml*), and streptomycin (100 *μg/ml*) in 37*°C* at 5% CO_2_. In the following, the cells were passaged using 0.25% trypsin, and the medium was refreshed every 3 days. Ascorbic acid (Vitamin C) was used as reference compound of angiogenesis inhibitor, in the following *in vitro* assays.

### MTT viability assay

To evaluate the cytotoxicity of the compound P and compound T, MTT assay was utilized in this research. In MTT assay, HUVEC cell line was treated with increasing doses of compounds P and T. To achieve this goal, HUVEC,10^4^ cells, were plated in 96-well flat-bottom plates and incubated for 24 *hr* at 37*°C* 7,28. Then, 50 *μl* of these compounds were added to each well of HUVEC cell line at concentrations rangs of 25, 50, and 100/*ml*. After 24 *hr* treatment, the medium was replaced with 100 *μl* of MTT [3-(4, 5-dimethylthiazol-2-yl]-2, 5-diphenyltetrazolium bromide) and was incubated for 4 *hr* in the dark conditions. After incubation, MTT solution was removed. Then, 100 *μl* of isopropanol was added to each well and sample was incubated for additional 30 *min* in the dark. In final, the reactive product was measured at 570 *nm* with a reference absorbance in 630 *nm* by using an ELISA reader (Organon Teknika, Netherlands). The experiments were repeated three times.

To obtaining IC_50_ for compound P and compound T, the cytotoxicity was calculated at different doses by the following formula:

$$\text{Cytotoxicity}=1-\frac{\text{Mean}\;\text{absorbance}\;\text{of}\;\text{toxicant}}{\text{Mean}\;\text{absorbance}\;\text{of}\;\text{negative}\;\text{control}}$$

In the following, Graphpad Instat software, version 3 was used to determine IC_50_ for compound P and compound T.

### Capillary-like tube formation assay

The *in vitro* tube formation assay was made to discover the VEGF neutralizing effects of compound P and compound T. In this assay, Matrigel was put at 4*°C* for overnight and then was diluted with equal volume of serum-free EBM-2 medium, for the final concentration of Matrigel as 5 *mg/ml*. Next, each well of 96-well plates were coated with 50 *μl* diluted Matrigel and were incubated at room temperature for 45 *min* 7,28. The starved HUVEC cells (1×10^4^ cells well^−1^) were plated in EBM-2 medium supplemented with 0.1% charcoal-stripped FBS, and then were treated with peptidomimetics at IC_50_ concentration (IC_50_ for compound T and compound P was 113 and 115 *μg/ml*, respectively) in the presence or absence of VEGF (50 *ng/ml*). VEGF and PBS were utilized as positive and negative control, respectively. Tubular structures were imaged and counted after 12 *hr* by Fluorescence microscopy (N800F model) under magnification 40X. The tubule structures in turn were scored by sprout formation counting.

### Quantitative real-time PCR

In the recent study, Quantitative Real-Time PCR was employed to determine whether the two compounds could inhibit the expression of *CD31* gene (endothelial cell marker) in HUVEC cells or not. *CD31* gene was selected because it was known as its expression is a marker of angiogenesis in HUVEC cells. Vitamin C and free angiogenesis inhibitor were used as the positive and negative controls. In this assay, total RNA extracted and purified by using RNeasy Plus mini kit (Qiagen, USA). The extracted RNA was diluted with 30 *μl* RNAse-free water and was quantified by spectrophotometer instrument (NanoDrop, Eppendorf, Germany). In the following, one-strand DNA was generated by using Prime Script RT Reagent Kit (TaKaRa, Japan). Gene Runner v. 3.05, Primer Express v. 2.5 and Beacon Designer v. 7.5 were used to design *CD31* gene primer. The primer sequences were included F: 5′-TCAAGCCTCAGCACCAGA-3′ and R: 5′-GCAC TCCTTCCACCAACAC-3′. Real-Time PCR was conducted through SYBR Premix Ex Taq II master mix (TaKaRa, Japan) on a one-step instrument (Applied Bio systems, USA). Specific primers for GAPDH, as endogenous control, were included F: 5′-GAGTCCAC TGGCGTCTTCA-3′ and R: 5′-TCTTGAGGCTGTT GTCATACTTC-3′. The thermal conditions for amplification were 95°*C* for 15S as holding time, 95°*C* for 5S and 60°*C* for 30S in each cycle. Fluorescence was collected in annealing-extension time in each cycle. Melting curve analysis was carried out in three steps: 95°*C*, 60°*C* and stepwise heated to 95°*C* with a ramp rate of 0.3°*C*. In final, data was analyzed by using ΔΔCt method.

### Statistical analyses

Graphpad Instat V3.00, was used for analyzing of data in MTT experiments. Comparison of groups was performed using t-test and one-way analysis of variance (ANOVA) by using SPSS V20.00. The results from the assays were expressed as the mean±SEM from three independent experiments. The level of statistical significance was set on 0.05.

## Results

### Collection of peptides and multiple sequence alignment

In the recent research, a total of 18 anti-VEGFR2 peptides were collected from previous studies (Table 1) 7,28–32. Multiple sequence alignment revealed RKRKKSR as the final peptide pattern. As seen in table 1, there are several arginine and lysine acid amines in this peptide pattern.

### Peptidomimetics design

The final peptide pattern, RKRKKSR, was obtained from 18 anti-angiogenic peptides that indicated more sequence homology to each other with high score (Table 2). The mimetic design-mediated pattern was used in a similarity search tool, namely PubChem, to find structures close to it. Finally, a lactam derivative [(2S, 5R, 6R)-3, 3-dimethyl-7-oxo-6-[(2-phenylacetyl) amino]-4-thia-1-azabicyclo [3.2.0] heptane-2-carboxylic acid] and an indole derivative [(2S)-2-amino-3-(1H-indol-3-yl) propanoic acid], were selected and used as compound P and compound T in this study (Figure 1).

**Table 2.** Two-way ANOVA of *CD31* gene expression in the negative and positive controls and samples treated with compound T and compound P

**Statistical analysis of gene expression**	**Significant difference**
Comparison of negative control samples with all concentrations of the two compounds	0.00
Comparison of positive control samples with all concentrations of the two compounds	0.00
Comparison of P with T at various concentrations of 50, 100 and 200	0.00

### MTT assay

**Figure 2.**
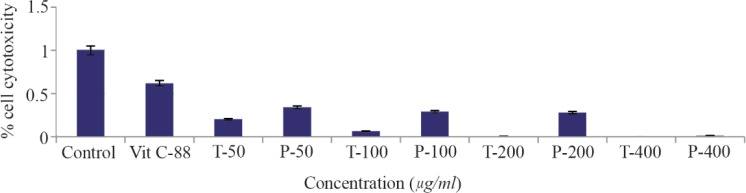
The results of MTT assay for compounds T and P by using HUVEC cells.HUVEC cells were treated with compounds T and P, at concentrations 25, 50, 100 *μg/ml* for 24 *hr*, and then cell cytotoxicity was evaluated with MTT assay. The IC_50_ for compound T and compound P in HUVEC cell line was 113 and 115 *μg/ml*, respectively. The bars represent the standard error for the mean of three replicates.

**Figure 3.**
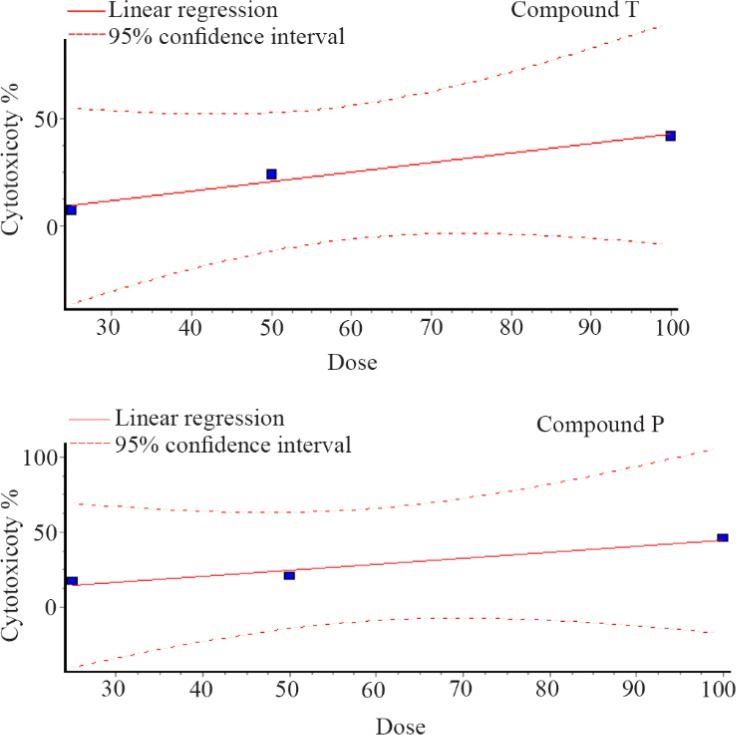
Dose response curve of MTT assay for compounds T and P, at different doses 25, 50, 100 *μg/ml*.

As shown in figure 2, the viability of the cells decreased after exposure to both compounds in a dose-dependent manner. The IC_50_ values for compound T and compound P in HUVEC cell line were 113 and 115 *μg/ml*, respectively. MTT assay showed a significant difference between T25 and control, T50 and control, T100 and control, P25 and control, P50 and control, as well as P100 and control. Dose response curves of MTT assay for compounds T and P, at different doses 25, 50, 100 *μg/ml* are presented in figure 3.

### Tube formation assay

**Figure 4.**
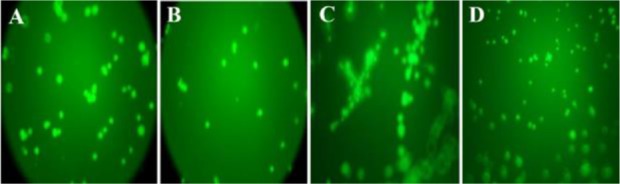
The level of decrease in sprout points for anti-angiogenesis compounds. The compound P and compound T were able to inhibit tube formation, as demonstrated by the decrease in sprout points in Matrigel (A and B). No significant effect was observed with negative control (PBS without VEGF) (D). VEGF treatment, as positive control, induces formation of capillary tube (C). The best inhibitory effect was demonstrated by compound T, where the level of decrease in sprout points was similar to VEGF-non treated cells (B).

As shown in figure 3, a decrease in the sprout points and tube formation was observed in VEGF-treated HUVEC after treatments with both compounds (Figure 4A and B). Tube formation was dependent on VEGF, where a network of tubes with several sprout points was more obvious in the VEGF-treated HUVEC than the VEGF-non-treated HUVEC (Figure 4C). Significant effect was not observed for negative control (PBS without VEGF) (Figure 4D). As a result, it was revealed that both compounds can inhibit angiogenesis, effectively.

### Real time PCR

**Figure 5.**
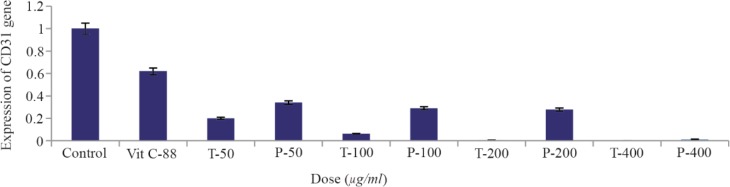
Effect of T compound and P compound on expression of *CD31* gene in HUVEC cell line using Real-Time PCR. Vitamin C (Vit C) and free-angiogenesis inhibitor Medium were used as positive and negative controls, respectively. The level of significant difference in the comparison of two compounds at concentrations of 50, 100, 200 was zero (p=0.00*). The level of significant level in comparison of positive control (vitamin C) and negative control with all the different concentrations of both compounds was zero (p=0.00*).

**Figure 6.**
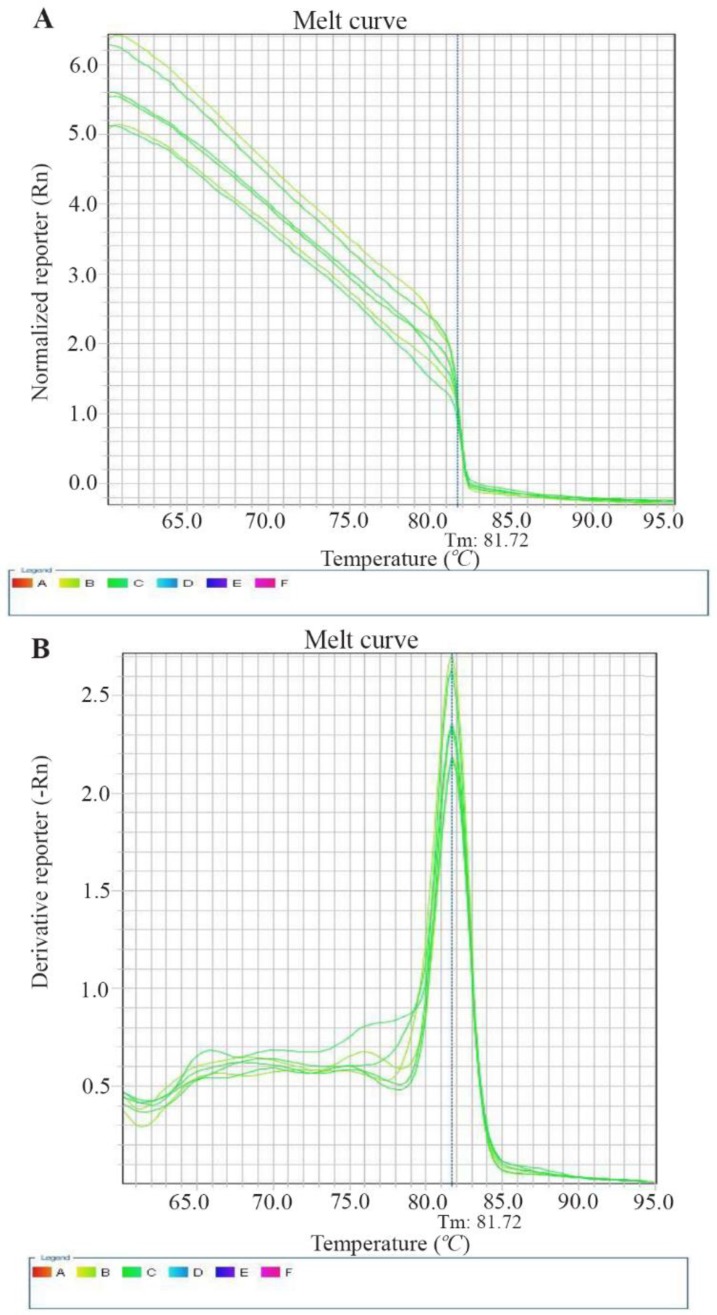
The melting curve of real-time PCR of *CD31* gene expression in the various concentrations of the two compounds P (A) and T (B).

Expression of *CD31* gene decreased dramatically at concentrations 50, 100, 200, 400 *μg/ml* of compound T in comparison to negative control (p<0.01), and positive control (p<0.05) (Figure 5, Table 2). Furthermore, it was observed a decrease in expression of *CD31* gene after treating cells with compound P in different doses, in comparison to negative and positive controls (p< 0.01). However, significant difference was not observed among concentrations 50, 100, 200 *μg/ml* of compound P (Figure 5). Interestingly, there is a significant difference (p<0.01) between both compounds in different concentrations (50, 100, 200 *μg/ml*) (Figure 5). The melting curve of real-time PCR of *CD31* gene expression in the various concentrations of the two compounds P (A) and T (B) is presented in figure 6.

The results of Real-Time PCR showed that, in all concentrations, the inhibitory effect of T compound on the CD31 expression is greater than that of P compound. We achieved some interesting results experimentally. In different doses of P and T, the expression of *CD31* gene decreased with increasing concentrations of both compounds. According to the statistical analysis, we conclude that T compound significantly reduced the expression of the *CD31* gene compared with the control sample (C vitamin). Regarding these points, T compound is a more suitable candidate for angiogenesis control.

## Discussion

In the cancer researches, VEGF is known as a key modulator for angiogenesis process. Thus, inhibiting the interaction VEGF and its receptor through antagonistic peptides is an effective and useful anti-angiogenic therapy 34. The studies on this item resulted in identification of important amino acids, which are involved in VEGF and its receptor interaction. Coupling this information with Alanine-scan analysis resulted in development of potent drug candidates with anti-angiogenic activity 34. Recently, researchers revealed the CPQPRPLC as a sequence targeting VEGFR1. In this way, the RPL shorter peptide was recognized as minimal sequence required for activity 35. In addition, it has been identified a peptide derived from *VEGF* gene, which involved in binding to HUVEC surface 36. This 20 amino acid peptide showed an inhibitory activity against cell migration and tumor growth in lung cancer.

In the current study, our efforts to design anti-angiogenic peptidomimetics resulted in finding two different compounds, namely P and T, by using in silico analysis 37. We first used MTT test to evaluate the cytotoxicity of the compounds. As a result of applying various concentrations of drugs, we detected 50% as 115 and 113 for compound P and compound T, respectively. We also conclude that these drug leads do not have a very toxic effect on HUVEC cells at certain doses. Therefore, these compounds can be considered as a treatment option, if they can inhibit target gene. In this regards, we used the results of this test to design real-time PCR. To do this test, according to real-time PCR analysis, it can be concluded that for concentrations above IC_50_ for compound T, there is no significant difference in inhibiting gene expression, because inhibition of gene expression has taken place completely in concentrations equal to or greater than IC_50_. By comparing the results of both compounds, we found that at all concentrations, T compound significantly reduced *CD31* gene expression compared with P compound. Comparison of gene expression in three different concentrations of each drug with negative control sample also showed that there was a significant difference in all concentrations in compared with negative control. Since toxicity of compound T is less, and also reduced *CD31* gene expression more, T compound seems to be more suitable candidate for angiogenesis control in cancers conditions.

Similar to our study, a number of researches have also reported some successes on design of anti-angiogenic peptidomimetics. Foy *et al* 38, for instance, designed peptidomemetics against VEGF. The authors indicated that peptidomemetics discovered can induce anti-tumor responses *in vitro* and *in vivo* 38. Foy *et al* 39 and Behelgardi *et al* 40 also demonstrated that the VEGF peptidomemetics can induce anti-angiogenic responses, effectively. Binétruy-Tournaire *et al* 16 screened a phage epitope library by affinity for an anti-VEGF neutralizing monoclonal antibody, and isolated peptides binding KDR specifically. Among synthetic peptides, ATWLPPR totally abolished VEGF binding to cell-displayed KDR. *In vitro*, this effect resulted in inhibition of VEGF-mediated proliferation of HUVECs in a dose-dependent manner, and endothelial cells in a type-specific manner. *In vivo*, ATWLPPR completely abolished VEGF-induced angiogenesis in rabbit model. As a result, the authors suggested ATWLPPR is an effective antagonist of VEGF binding, and this peptide can be a suitable inhibitor of tumor angiogenesis 16. In agreement with our findings, their results revealed that designing of anti-angiogenic peptidomimetics by using SuperMimic, and evaluation their biological activity by *in vitro* assays can be used to find inhibitors of tumor angiogenesis.

Vicari *et al*, designed peptides to mimic VEGF-binding site to its receptor VEGFR-2. The VEGF peptide mimic, VEGF-P3 (CYC), showed the highest affinity to VEGFR-2 by surface plasmon resonance assay. In addition, in several angiogenic in vitro assays, the authors observed that all VEGF mimics inhibited endothelial cell proliferation, migration, and network formation with the conformational VEGF-P3 (CYC) being the best. Similarly, their findings demonstrated that the structure-based design is important for development of anti-angiogenic peptidomimetics 10. Kim *et al*, 28 also developed MAP2-dRK6 peptide with high anti-tumor and anti-VEGF activity. MAP2-dRK6 peptide was effective in many respects- such as inhibition of VEGF binding to its receptors, VEGF-induced migration, ERK signaling of endothelial cells, and tube formation of endothelial cells. In addition, MAP2-dRK6 blocks growth of VEGF-secreting colorectal cancer cells *in vivo* by suppression of angiogenesis 28. In agreement with our findings, their results revealed that anti-angiogenic peptidomimetics can be used as lead compound or therapeutic molecule for development of drugs for VEGF-related angiogenic diseases 41,42.

## Conclusion

Since, angiogenesis is severely influenced by proangiogenic VEGF, promising anti-angiogenic drugs are important and currently available; however, their susceptibilities to drug resistance and long term toxicity are serious obstacles to their use. As a result, we require the development of novel therapeutic approaches for effective and safe angiogenic inhibitors. We designed anti-VEGFR peptidomimetics and evaluated their biological activity by *in vitro* assays. Compounds P and T inhibited HUVEC cells proliferation and viability in a dose-dependent manner. The IC_50_ for compound T and compound P in HUVEC cell line was 113 and 115 *μg/ml*, respectively. Tube formation assay revealed that both compounds can inhibit angiogenesis effectively. The results of Real-Time PCR also showed these compounds are able to inhibit the expression of *CD31* gene in HUVEC cell line. In summary, our results revealed that compound P (lactam derivative) and compound T (indole derivative) have anti-angiogenic activity and can be used for further studies in order to discovery and design anti-angiogenesis compounds.
